# Endovascular closure of a large intrahepatic portosystemic shunt in a three-month-old infant: a case report and literature review

**DOI:** 10.3389/fped.2026.1862205

**Published:** 2026-06-17

**Authors:** Konstantin Semash, Shukhrat Salakhitdinov, Mansur Nasirov, Timur Dzhanbekov, Alisher Yusubov, Bakhtiyorjon Umarov

**Affiliations:** Department of Minimally Invasive Surgery and Transplantation, National Children’s Medical Center, Tashkent, Uzbekistan

**Keywords:** case report, congenital portosystemic shunt, endovascular embolization, infant, intrahepatic shunt

## Abstract

**Introduction:**

Congenital portosystemic shunts (CPSS) are rare vascular anomalies characterized by partial or complete diversion of portal blood into the systemic circulation, bypassing hepatic filtration. Early recognition and timely intervention are crucial to prevent severe metabolic and developmental complications.

**Case presentation:**

We report a rare case of a three-month-old male infant presenting with persistent jaundice and growth retardation. Laboratory tests revealed marked hyperbilirubinemia (316 µmol/L) and elevated transaminase levels (ALT 944 U/L, AST 405 U/L). Doppler ultrasonography and contrast-enhanced CT demonstrated a large intrahepatic portosystemic shunt (up to 25 mm in diameter) with hypoplasia of the right hepatic lobe. Following multidisciplinary team discussion, endovascular coil embolization was performed via a transjugular approach. An early postoperative complication occurred due to coil migration into the pulmonary artery, which was successfully managed with endovascular retrieval. The patient showed rapid normalization of liver function tests, restoration of portal blood flow, and catch-up growth during follow-up.

**Conclusion:**

This case highlights the feasibility, safety, and effectiveness of endovascular closure of large intrahepatic CPSS even in very young infants. It underscores the critical importance of thorough anatomical evaluation, multidisciplinary planning, and meticulous postoperative monitoring to ensure optimal outcomes and to promptly address potential complications.

## Introduction

Congenital portocaval shunts (CPS), also referred to as congenital portosystemic shunts (CPSS), are rare vascular anomalies characterized by an abnormal connection allowing partial or complete diversion of portal venous blood directly into the systemic circulation, thereby bypassing the liver (1). These malformations include both extrahepatic and intrahepatic variants and result from aberrations in embryological development, specifically involving incomplete regression or persistence of venous communications between the portal and systemic venous systems (2).

The clinical presentation of CPSS is highly variable, ranging from asymptomatic cases discovered incidentally to severe manifestations such as hyperammonemia, hepatic encephalopathy, portal hypertension, hepatic hypoplasia, and hepatopulmonary syndrome. In many instances, these shunts are identified incidentally during imaging studies performed for unrelated reasons (3). Diagnosis relies on a combination of Doppler ultrasonography, contrast-enhanced computed tomography (CT), or magnetic resonance imaging (MRI), with selective portography often serving as a critical tool for detailed delineation of the shunt anatomy and portal vein architecture (4).

Current therapeutic options encompass both endovascular and surgical approaches. The choice of treatment is guided by anatomical characteristics of the shunt, the degree of preserved portal perfusion, and the patient's overall clinical status (5, 6). Endovascular closure has emerged as a minimally invasive and highly effective alternative to open surgical repair in patients with a patent portal vein and adequate hepatic parenchymal reserve (6).

## Case presentation

This case report was written following the CARE (CAse REport) guidelines to ensure standardized and transparent reporting. Written informed consent was obtained from the patient's parents for publication of this case. Additionally, this study was approved by the institutional review board (IRB Statement No. 3611-77, dated July 8, 2025).

**Figure 1:**
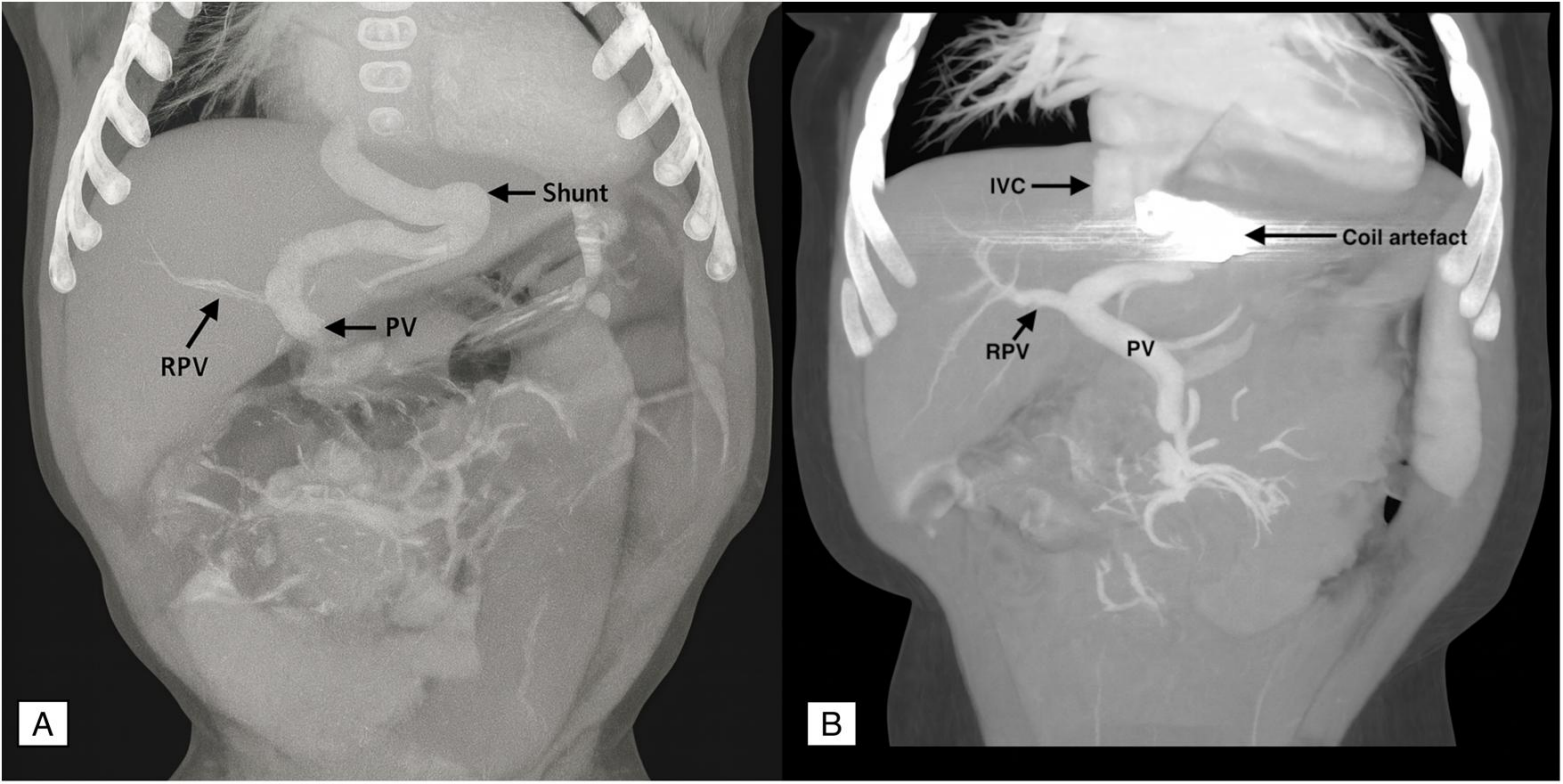
Contrast-enhanced computed tomography. **(A)** Initial examination revealed a large intrahepatic shunt originating from the left portal vein (PV) branch. The right portal vein branch (RPV) appeared hypoplastic. **(B)** Follow-up examination 6 months postoperatively demonstrated improved caliber and filling of the right portal vein branch following restoration of hepatopetal portal flow. No residual flow through the shunt was detected. A coil artefact is visible at the embolization site.

A three-month-old male infant was referred to our center with complaints of persistent jaundice and delayed physical development. The infant was born at 37 weeks of gestation with a birth weight of 3.1 kg. At the time of admission, his body weight was 5.2 kg. The patient was evaluated using our internal protocol for liver disease assessment. Laboratory investigations revealed hyperbilirubinemia (total bilirubin level 316 μmol/L) and elevated transaminase levels (ALT U/L 944 and AST 405 U/L). Doppler ultrasonography demonstrated an abnormal blood shunt from the portal system to the inferior vena cava. Contrast-enhanced computed tomography (CT) identified a large intrahepatic CPSS measuring up to 25 mm in diameter (Figure 1A). Blood flow in the right branch of the portal vein was significantly reduced, and the right hepatic lobe appeared hypoplastic.

Given the clinical presentation and imaging findings, a comprehensive literature review was conducted, and a multidisciplinary decision was made to perform endovascular closure of the shunt. Prior to the intervention, the parents were thoroughly counseled regarding the potential risks and benefits of the procedure. They were informed that possible intra- and postoperative complications could include portal vein thrombosis, coil migration, or acute portal hypertension. The family was also advised that if endovascular closure proved technically unfeasible, open surgical ligation might be required, and in case of an unfavorable outcome, liver transplantation could become necessary. After comprehensive discussion with the multidisciplinary team, the parents gave written informed consent for surgery.

**Figure 2:**
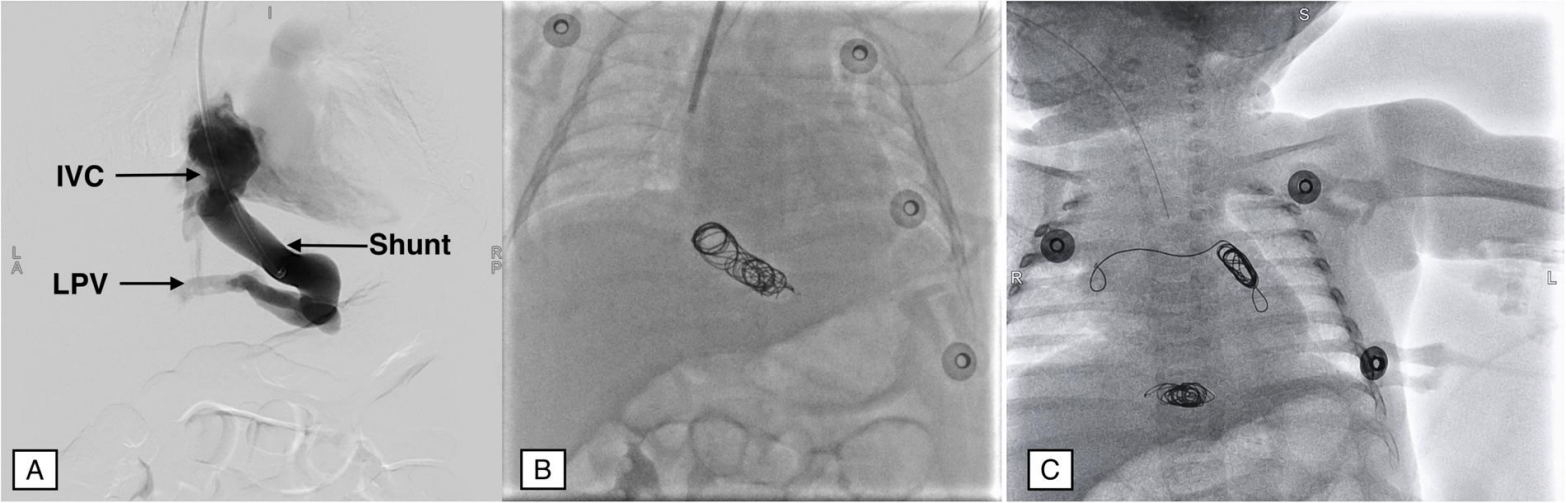
Intraoperative fluoroscopy during endovascular intervention. **(A)** A large intrahepatic shunt is visualized, originating from the left portal vein branch (LPV) and draining into the inferior vena cava (IVC). **(B)** Final appearance after placement of coils within the shunt lumen. **(C)** Intraoperative fluoroscopic image demonstrating migration of the 20 mm × 40 cm coil into the pulmonary artery during endovascular embolization of the congenital intrahepatic portosystemic shunt. The two 16 mm × 30 cm anchoring coils remained stably positioned within the shunt.

During the surgery, a cavoportogram via transjugular access revealed a large intrahepatic CPSS measuring 26 mm in diameter and approximately 65 mm in length, with communication to the left branch of the portal vein (Figure 2A). No evidence of additional accessory shunts or multifocal intrahepatic communications was observed.

The procedure was technically challenging because of the patient's young age, small vessel caliber, and the large size of the shunt relative to the infant's vascular anatomy. Transjugular access was selected to provide the most direct and stable route to the shunt while minimizing the risk of vascular injury. Careful catheter manipulation and continuous ultrasound and fluoroscopic guidance were required throughout the procedure due to the limited vascular dimensions.

Direct intraoperative portal pressure measurement and balloon occlusion testing were not performed because a balloon catheter of the required diameter could not be safely advanced through the very small right internal jugular vein. Given these anatomical limitations, endovascular coil embolization was selected as the safest strategy. Unlike vascular plug occlusion, coils induce progressive thrombosis and gradual shunt closure over weeks to months, thereby potentially minimizing abrupt increases in portal venous pressure and reducing the risk of acute portal hypertension. The procedure was carried out under continuous hemodynamic and Doppler ultrasound monitoring, confirming preserved intrahepatic portal perfusion throughout and after embolization.

Embolization was performed using three detachable coils (Concerto™, Medtronic, USA): one 20 mm  ×  40 cm coil and two 16 mm × 30 cm coils. Because of the extremely small caliber of the infant's internal jugular vein, the 20 mm device represented the largest coil that could be safely advanced through the available vascular access. The two 16 mm coils were initially deployed proximally as anchoring coils to create a stable framework and reduce the risk of migration within the high-flow shunt. Subsequently, the 20 mm coil was deployed as the primary occlusion device to promote progressive thrombosis of the shunt lumen (Figure 2B).

Postoperatively, the patient was monitored in the intensive care unit. The immediate postoperative course was stable; however, follow-up chest radiography raised suspicion of coil displacement. The patient was returned to the catheterization laboratory, where angiography confirmed migration of the 20 mm coil into the pulmonary artery, whereas both 16 mm anchoring coils remained stably positioned without displacement (Figure 2C). The migrated coil was successfully retrieved using an endovascular snare system (Merit Medical, USA), although retrieval was technically challenging because of the high-flow hemodynamics within the pulmonary circulation.

The postoperative regimen included ursodeoxycholic acid (10 mg/kg/day) and low-dose aspirin (3 mg/kg/day) for thromboprophylaxis. The choice of antiplatelet therapy was based on maintained hepatopetal portal flow on Doppler imaging and the absence of thrombotic risk factors. Doppler surveillance was performed on postoperative days 1 and 7, and at 1, 3, and 6 months. Portal vein diameter and mean velocity were 3.5 mm and 21 cm/s pre-closure, 4.2 mm and 28 cm/s at 1 month, and 4.8 mm and 32 cm/s at 6 months, respectively. Escalation to low-molecular-weight heparin (enoxaparin 1 mg/kg twice daily) was planned if portal flow decreased below 20 cm/s or thrombus formation was detected; however, this was not required. No evidence of portal vein thrombosis or flow reduction was observed during follow-up.

At the one-month follow-up, the patient demonstrated a decrease in bilirubin levels to 50 µmol/L, normalization of liver enzyme levels, and improved portal blood flow to the right portal vein branch (Table 1). At the three-month outpatient follow-up, all biochemical parameters remained within normal limits. Six months postoperatively, contrast-enhanced multidetector CT revealed restoration of portal blood flow with improved filling of the right portal vein (Figure 1B). Quantitative CT volumetry at 6 months showed right hepatic lobe volume growth from 35 cm^3^ to 46 cm^3^, demonstrating interval increase in right hepatic lobe volume following restoration of portal perfusion. Anthropometric follow-up demonstrated steady weight gain from below the 5th to the 50th percentile, with normal developmental milestones for age. Laboratory values remained normal. Serial Doppler ultrasound examinations demonstrated preserved hepatopetal portal flow, progressive increase in portal vein diameter and velocity, and no evidence of thrombosis or portal hypertension during follow-up. In addition, the patient did not develop clinical signs suggestive of portal hypertension, including ascites, splenomegaly, or gastrointestinal bleeding.

**Table 1:** Laboratory and Doppler findings during follow-up.

Parameter	Baseline	1 month	3 months	6 months
Total bilirubin (µmol/L)	316	50	38	15
ALT (U/L)	944	85	42	38
AST (U/L)	405	60	38	36
Portal vein diameter (mm)	3.5	4.2	4.5	4.8
Mean portal velocity (cm/s)	21	28	30	32
Weight (kg)	5.2	6.1	7.0	8.2
Length (cm)	58	61	64	69
WHO percentile (weight-for-age)	<5th	10th	25th	60th

One year after surgery, the child demonstrates age-appropriate development (Table 2). According to the parents’ report, they were highly satisfied with the treatment and overall outcome.

**Table 2:** Timeline of clinical course and management.

Time point	Clinical event
3 months of age	Persistent cholestasis, impaired liver function, poor weight gain
Diagnostic imaging	Large intrahepatic portal–IVC shunt with reduced portal perfusion identified on Doppler US and CT
Multidisciplinary discussion	Decision for endovascular closure
Procedure day	Transjugular coil embolization performed
Intraoperative complication	Migration of 20 mm coil into pulmonary artery
Second procedure	Successful snare retrieval of migrated coil
Early postoperative period	Preserved hepatopetal portal flow and stable condition
1–3 months follow-up	Progressive biochemical improvement
6 months follow-up	Improved portal perfusion and hepatic growth on CT
Long-term follow-up	Favorable clinical outcome without portal hypertension

## Discussion

CPSS is a rare developmental vascular anomaly characterized by partial or complete diversion of portal venous blood into the systemic circulation, bypassing the liver (5). This abnormal circulation allows unfiltered blood from the intestines to enter the systemic circulation, leading to various clinical manifestations including hyperammonemia, hepatic encephalopathy, growth retardation, and pulmonary complications. CPSS arises in utero due to disturbances in the embryological development of the portal venous system (7).

The first case of CPSS was described by John Abernethy in 1793, who identified direct drainage of the portal vein into the inferior vena cava in a 10-month-old infant during autopsy (8). Such malformations, particularly those with complete absence of intrahepatic portal perfusion, have been termed “Abernethy malformations” in subsequent literature.

Initially, CPSS was reported in isolated case studies during the mid-20th century. Beginning in the 1970s, surgical ligation procedures were described, and by the 1990s, endovascular techniques and laparoscopic ligation began to be adopted. Over the last two decades, larger case series have been published, including reports of 15–20 patients, with the largest single-center cohort encompassing up to 40 patients. As of 2022, approximately 700 cases have been described worldwide.

Several classifications have been proposed to guide diagnosis and treatment (Table 3). Morgan and Superina first divided CPSS into two main types based on intrahepatic portal perfusion: Type I, with complete absence of portal perfusion, and Type II, with partial preservation (9). In 2008, Stringer suggested dividing shunts into intrahepatic and extrahepatic variants (10). In 2011, Lautz et al. further refined Type II into subtypes IIa (ductus venosus-like), IIb (originating from the main portal vein near the splenomesenteric confluence and bifurcation), and IIc (arising from mesenteric, gastric, or splenic veins), with Type I patients considered candidates for liver transplantation (11). Blanc et al. proposed a surgical classification based on the anatomical fusion between the portal and systemic venous systems to guide operative management (12). Later, Kanazawa et al. added an angiographic component, incorporating intrahepatic portal vein hypoplasia severity and balloon occlusion testing to identify patients at higher postoperative risk (13). In our case, hemodynamic testing with balloon occlusion could not be performed because of technical limitations related to the infant's small venous caliber. The gradual thrombogenic mechanism of coil embolization allowed a physiologic redistribution of portal flow and avoided a sudden rise in portal pressure. This strategy is consistent with the principle of staged or progressive closure recommended in patients with high-flow, large-caliber shunts or limited intrahepatic portal development.

**Table 3:** Classification of congenital portosystemic shunts.

Author	Type of shunt	Treatment approach
Morgan & Superina (9)	Type I: complete absence of portal liver perfusion Type II: partial portal perfusion	Type I: orthotopic liver transplantation Type II: endovascular closure or surgical correction (if symptomatic or complicated)
Lautz et al. (11)	Type I: no intrahepatic portal flow. Type II: partial shunt with preserved hepatic flow: • IIa: shunt arising from the left or right portal vein (includes persistent ductus venosus). • IIb: shunt arising from the main portal vein (including its bifurcation or splenomesenteric confluence). • IIc: shunt arising from mesenteric, gastric, or splenic veins.	Type I: orthotopic liver transplantation Type II: endovascular embolization (if technically feasible); if ineffective — surgical intervention
Blanc et al. (12)	Type I: complete absence of portal perfusion (all blood diverted into the systemic circulation). Type II: partial portal perfusion, subdivided into: • IIa: extrahepatic end-to-side shunt. • IIb: extrahepatic side-to-side shunt. • IIc: intrahepatic shunt. Type III: portohepatic pattern. Type IV: persistent ductus venosus pattern.	Type I: orthotopic liver transplantation Types II–IV: endovascular or surgical closure (a staged approach may be considered for large shunts)
Kanazawa et al. (13)	Mild type: well-developed intrahepatic portal system. Moderate type: moderately developed intrahepatic portal system. Severe type: poorly developed or absent intrahepatic portal system.	Mild type: one-step closure feasible. Moderate type: one-step closure usually possible with monitoring. Severe type: staged closure or liver transplantation

Although spontaneous closure of small intrahepatic CPSS has been reported during early childhood, particularly within the first two years of life, early intervention is generally recommended in symptomatic patients or in cases associated with significant metabolic, hemodynamic, or hepatic abnormalities (14, 15). In the present case, the patient demonstrated severe hyperbilirubinemia, markedly elevated transaminases, impaired portal perfusion, right hepatic lobe hypoplasia, and delayed physical development. These findings suggested clinically significant shunt physiology with ongoing hepatic dysfunction, which was considered a clear indication for active intervention rather than expectant observation.

Endovascular embolization has emerged as a preferred modality due to its minimally invasive nature, avoidance of laparotomy-related complications, shorter hospitalization, and favorable postoperative recovery, which is especially important in infants and young children (14). Endovascular treatment of CPSS in early infancy presents unique technical challenges related to small vascular caliber, limited device compatibility, and high-flow hemodynamics. In low-weight infants, vascular access itself may become a limiting factor, particularly when large delivery systems or balloon occlusion catheters are required. Careful procedural planning and device selection are therefore critical to minimize vascular injury and procedural complications. Nevertheless, endovascular closure may become technically unfeasible in patients with extremely complex vascular anatomy, multifocal shunts, severely hypoplastic intrahepatic portal veins, unfavorable landing zones for device fixation, or inability to safely obtain adequate vascular access. In such situations, staged surgical ligation or liver transplantation may need to be considered.

Although endovascular closure of CPSS in infants as young as 3–6 months has been reported (7, 14). This case is distinctive for the unusually large shunt caliber (26 mm × 65 mm), the occurrence and successful percutaneous retrieval of coil migration. However, complications such as coil migration, portal vein thrombosis, and transient portal hypertension have been reported, emphasizing the need for meticulous technique and rigorous postoperative monitoring (15–18). Coil migration remains one of the most serious technical complications during embolization of large high-flow CPSS, particularly in infants with limited vascular access and unfavorable shunt geometry (17). In the present case, several factors likely contributed to migration of the 20 mm coil, including persistent high-flow shunt hemodynamics, the large diameter and length of the shunt, limited landing zone stability, and technical restrictions related to device selection in a very small infant. Although two 16 mm anchoring coils remained stably positioned, the larger 20 mm coil likely experienced insufficient fixation within the high-flow vascular channel before stable thrombosis formation occurred. This complication emphasizes several important technical considerations for future procedures. First, secure proximal anchoring is critical in large-caliber shunts. Second, staged embolization or vascular plug-assisted techniques may theoretically improve stability in selected patients. Third, device selection in infants is frequently constrained by vascular access caliber and delivery system compatibility. Finally, immediate postoperative imaging surveillance is essential for early detection and prompt management of migration-related complications. Importantly, despite migration into the pulmonary artery, the coil was successfully retrieved endovascularly using a snare system without residual clinical sequelae. Several preventive strategies have been proposed in the literature, including the use of oversized framing coils, creation of stable proximal anchoring zones, staged embolization approaches, vascular plug-assisted techniques, and combined coil-plug constructs in selected patients (14–18). However, in very small infants, device selection is frequently constrained by vascular access caliber and delivery system compatibility. In our patient, despite deployment of two anchoring coils, persistent high-flow hemodynamics likely contributed to migration of the larger occlusion coil before stable thrombosis formation occurred.

Compared with previously published pediatric CPSS series (Table 4), our case represents a uniquely challenging technical scenario because standard balloon occlusion testing and direct portal pressure measurement could not be performed due to the extremely small caliber of the infant's internal jugular vein. Most published reports rely on balloon occlusion and portal pressure thresholds to guide management decisions (19–24). In contrast, our treatment strategy was based on detailed angiographic assessment, continuous Doppler monitoring.

**Table 4:** Comparative overview of published congenital portosystemic shunt cases: hemodynamic assessment, treatment strategies, and clinical outcomes.

Study	N	Age	CPSS type	Main presentation	Hemodynamic assessment	Treatment	Devices/materials	Complications	Outcome
Robinson et al. (19)	5	Neonates–children	Mainly intrahepatic	Cholestasis, hyperammonemia, pulmonary HTN, hypoglycemia, hepatic lesions	Balloon occlusion and portal pressure when feasible	Endovascular or staged surgery	Detachable coils, Amplatzer devices	None major reported	Good clinical recovery after tailored management
Koneti et al. (20)	24	0.5–21 years	Mainly extrahepatic	PAVM, PH, respiratory distress	Balloon occlusion mandatory; closure if portal pressure ≤18 mmHg	Transcatheter closure	AVP II, septal occluders, muscular devices, stent grafts	Some patients unsuitable because of portal HTN	Successful closure in 21/24
Tiwari et al. (21)	3	Pediatric/young adult	Intra- and extrahepatic	PH, hepatopulmonary syndrome, hyperammonemia	Balloon occlusion and portal pressure monitoring	Endovascular closure	VSD devices, plugs, Konar MFO	No major procedural complications	Significant symptomatic improvement
Tran et al. (22)	21	Pediatric	Intra- and extrahepatic	Cholestasis, hyperammonemia, liver masses, pulmonary complications	One-stage closure considered if portal pressure <30 mmHg	Observation, surgery, endovascular closure	Various devices	No major complications reported	Good outcomes after intervention
McLin et al. (15)	Expert consensus	Pediatric/adult	Intra- and extrahepatic	Multisystem complications	Occlusion test regarded as gold standard	Endovascular, surgery, LT	Tailored approach	Discussed risk of portal HTN and liver tumors	Consensus recommendations
DiPaola et al. (7)	11	Children/young adults	Intra- and extrahepatic	Hyperammonemia, HPS, pulmonary HTN, liver tumors	Angiographic evaluation	Endovascular, surgery, LT	Multiple techniques	HCC and adenomas reported	Good long-term outcomes after intervention
Bayona Molano et al. (16)	2	Adults	Intrahepatic	Encephalopathy	Balloon occlusion test	Endovascular embolization	Embolization coils	No major complications reported	Resolution of symptoms
Oktay et al. (24)	1	Neonate	Intrahepatic	Neonatal cholestasis	Ultrasound monitoring only	Conservative	None	None	Spontaneous closure
Semash et al. (Present case)	1	3-month-old infant	Intrahepatic	Severe cholestasis, impaired portal perfusion, hepatic hypoplasia	Direct portal pressure measurement and balloon occlusion impossible because of extremely small jugular vein caliber	Transjugular endovascular coil embolization	Three detachable Concerto™ coils (20 mm × 40 cm and two 16 mm × 30 cm)	Migration of 20 mm coil into pulmonary artery requiring technically difficult snare retrieval	Successful retrieval, preserved portal perfusion, progressive hepatic growth and favorable clinical outcome

In previously published reports from our center, we have highlighted that, despite functioning as a tertiary referral hospital, resource limitations remain an important factor influencing complex endovascular and transplant procedures in our region. In particular, vascular plugs and large-caliber delivery systems are not always consistently available in our institution (25). Nevertheless, treatment strategy in each case is primarily determined by technical feasibility, anatomical considerations, and careful risk–benefit assessment. In the present patient, we additionally considered gradual coil-induced thrombosis potentially advantageous compared with immediate complete plug occlusion, as abrupt closure of a large high-flow shunt may theoretically result in sudden portal venous pressure elevation and acute portal hypertension. Progressive shunt thrombosis following coil embolization may allow more gradual adaptation of the portal venous system to restored hepatopetal flow.

Post-embolization antithrombotic management after CPSS closure in infants remains non-standardized, and current practice is largely individualized according to portal flow dynamics, thrombosis risk, and institutional experience (15, 26). Doppler ultrasonography remains the primary modality for surveillance, and anticoagulation therapy should be initiated if portal flow decreases significantly (17). In our case, single-agent aspirin was considered sufficient given preserved hepatopetal flow and stable Doppler parameters. The gradual nature of coil-induced occlusion and serial Doppler follow-up likely contributed to smooth hemodynamic adaptation without need for escalation to heparin therapy. In our patient, preserved hepatopetal portal flow, stable Doppler parameters, and the absence of radiological evidence of thrombosis supported the use of aspirin monotherapy combined with close ultrasound surveillance. Escalation to therapeutic anticoagulation with low-molecular-weight heparin was predefined in case of portal flow reduction or thrombus formation. This stepwise strategy allowed individualized thromboprophylaxis while minimizing the bleeding risks associated with systemic anticoagulation in early infancy.

This report has several limitations, including its single-patient design, absence of direct portal pressure measurements and balloon occlusion testing, and limited generalizability of technical decisions because of infant vascular anatomy and institution-specific resource constraints. Nevertheless, the case provides important technical insights into management of large high-flow CPSS in very young infants.

This case highlights the importance of individualized, anatomy-driven treatment planning, emphasizes the feasibility and safety of endovascular approaches even in infants, and underscores the necessity for careful postoperative surveillance to prevent and promptly manage complications.

## Conclusion

Congenital portosystemic shunts represent a rare but potentially severe vascular anomaly requiring precise anatomical assessment and individualized management. Our case demonstrates that endovascular coil embolization is a feasible, effective, and minimally invasive treatment option even in very young infants, provided that portal vein patency and adequate intrahepatic perfusion are confirmed. Successful closure can result in rapid biochemical normalization, improvement of portal perfusion, and age-appropriate development. Rigorous postoperative monitoring remains essential to ensure optimal outcomes and timely management of complications.

## Data Availability

The original contributions presented in the study are included in the article/Supplementary Material, further inquiries can be directed to the corresponding author.
